# Supplementary material for: Multi‐dataset identification of innovative feature genes and molecular mechanisms in keratoconus
Source: J Cell Mol Med. 2024 Sep 19;28(18):e70079. doi: 10.1111/jcmm.70079 (PMC11412914; doi:10.1111/jcmm.70079)
Supplement: Supplementary file 2 — Material S2. [file JCMM-28-e70079-s002.zip › 7.GSEA analysis/2.ATOH7/KEGG_TYPE_II_DIABETES_MELLITUS.html]

Details for gene set KEGG\_TYPE\_II\_DIABETES\_MELLITUS[GSEA]

|  || Dataset | ATOH7.group.cls#h\_versus\_l |
| Phenotype | group.cls#h\_versus\_l |
| Upregulated in class | h |
| GeneSet | KEGG\_TYPE\_II\_DIABETES\_MELLITUS |
| Enrichment Score (ES) | 0.5245108 |
| Normalized Enrichment Score (NES) | 1.5955385 |
| Nominal p-value | 0.030947777 |
| FDR q-value | 0.35935497 |
| FWER p-Value | 0.608 |
Table: GSEA Results Summary

  

Fig 1: Enrichment plot: KEGG\_TYPE\_II\_DIABETES\_MELLITUS      
 Profile of the Running ES Score & Positions of GeneSet Members on the Rank Ordered List

  

| SYMBOL | TITLE | RANK IN GENE LIST | RANK METRIC SCORE | RUNNING ES | CORE ENRICHMENT || 1 | IRS1 | na | 55 | 0.848 | 0.0994 | Yes |
| 2 | SOCS2 | na | 89 | 0.782 | 0.1917 | Yes |
| 3 | SLC2A2 | na | 608 | 0.518 | 0.2386 | Yes |
| 4 | CACNA1G | na | 646 | 0.512 | 0.2985 | Yes |
| 5 | CACNA1B | na | 672 | 0.503 | 0.3577 | Yes |
| 6 | KCNJ11 | na | 797 | 0.479 | 0.4112 | Yes |
| 7 | PRKCE | na | 1002 | 0.447 | 0.4587 | Yes |
| 8 | CACNA1E | na | 1688 | 0.370 | 0.4829 | Yes |
| 9 | GCK | na | 1752 | 0.364 | 0.5245 | Yes |
| 10 | IRS2 | na | 3662 | 0.249 | 0.4992 | No |
| 11 | SLC2A4 | na | 4197 | 0.229 | 0.5111 | No |
| 12 | HK2 | na | 5782 | 0.177 | 0.4865 | No |
| 13 | SOCS3 | na | 5926 | 0.173 | 0.5030 | No |
| 14 | PIK3R3 | na | 6166 | 0.166 | 0.5159 | No |
| 15 | CACNA1A | na | 8517 | 0.108 | 0.4610 | No |
| 16 | SOCS1 | na | 9819 | 0.084 | 0.4335 | No |
| 17 | PKM | na | 10652 | 0.071 | 0.4180 | No |
| 18 | HK1 | na | 11153 | 0.064 | 0.4112 | No |
| 19 | INS | na | 11499 | 0.059 | 0.4083 | No |
| 20 | HK3 | na | 13130 | 0.039 | 0.3659 | No |
| 21 | ADIPOQ | na | 13485 | 0.035 | 0.3599 | No |
| 22 | TNF | na | 14399 | 0.027 | 0.3368 | No |
| 23 | MAPK1 | na | 14536 | 0.025 | 0.3359 | No |
| 24 | PIK3CD | na | 16202 | 0.011 | 0.2892 | No |
| 25 | MAPK3 | na | 16821 | 0.006 | 0.2721 | No |
| 26 | MAPK8 | na | 17140 | 0.003 | 0.2633 | No |
| 27 | PRKCD | na | 17924 | -0.003 | 0.2412 | No |
| 28 | SOCS4 | na | 18050 | -0.004 | 0.2381 | No |
| 29 | INSR | na | 18814 | -0.011 | 0.2174 | No |
| 30 | MAFA | na | 19255 | -0.015 | 0.2065 | No |
| 31 | PIK3CB | na | 20685 | -0.028 | 0.1687 | No |
| 32 | MAPK10 | na | 20989 | -0.032 | 0.1638 | No |
| 33 | PRKCZ | na | 21192 | -0.034 | 0.1620 | No |
| 34 | PIK3CA | na | 21359 | -0.036 | 0.1614 | No |
| 35 | PDX1 | na | 21668 | -0.039 | 0.1572 | No |
| 36 | CACNA1C | na | 22079 | -0.044 | 0.1506 | No |
| 37 | PIK3R2 | na | 22209 | -0.045 | 0.1523 | No |
| 38 | IRS4 | na | 23255 | -0.058 | 0.1291 | No |
| 39 | IKBKB | na | 23356 | -0.060 | 0.1333 | No |
| 40 | MAPK9 | na | 24826 | -0.082 | 0.1008 | No |
| 41 | MTOR | na | 25211 | -0.089 | 0.1003 | No |
| 42 | PIK3CG | na | 26210 | -0.109 | 0.0845 | No |
| 43 | CACNA1D | na | 26374 | -0.112 | 0.0932 | No |
| 44 | PIK3R1 | na | 27720 | -0.144 | 0.0715 | No |
| 45 | PKLR | na | 30222 | -0.219 | 0.0255 | No |
| 46 | ABCC8 | na | 31416 | -0.265 | 0.0226 | No |
| 47 | PIK3R5 | na | 34383 | -0.611 | 0.0099 | No |
Table: GSEA details [plain text format]

  

Fig 2: KEGG\_TYPE\_II\_DIABETES\_MELLITUS      
 Blue-Pink O' Gram in the Space of the Analyzed GeneSet

  

Fig 3: KEGG\_TYPE\_II\_DIABETES\_MELLITUS: Random ES distribution      
 Gene set null distribution of ES for **KEGG\_TYPE\_II\_DIABETES\_MELLITUS**
